# Methodological Problems on the Way to Integrative Human Neuroscience

**DOI:** 10.3389/fnint.2016.00041

**Published:** 2016-11-29

**Authors:** Boris Kotchoubey, Felix Tretter, Hans A. Braun, Thomas Buchheim, Andreas Draguhn, Thomas Fuchs, Felix Hasler, Heiner Hastedt, Thilo Hinterberger, Georg Northoff, Ingo Rentschler, Stephan Schleim, Stephan Sellmaier, Ludger Tebartz Van Elst, Wolfgang Tschacher

**Affiliations:** ^1^Institute of Medical Psychology and Behavioral Neurobiology, University of TübingenTübingen, Germany; ^2^Bertalanffy Centre for the Study of Systems ScienceVienna, Austria; ^3^Bavarian Academy for Addiction and Health Issues (BAS)Munich, Germany; ^4^AG Neurodynamics, Institute of Physiology and Pathophysiology, Philipps University of MarburgMarburg, Germany; ^5^Department of Philosophy I, Ludwig Maximilian University of MunichMunich, Germany; ^6^Institute of Physiology and Pathophysiology, Faculty of Medicine, University of HeidelbergHeidelberg, Germany; ^7^Department of General Psychiatry, Centre of Psychosocial Medicine, University of HeidelbergHeidelberg, Germany; ^8^Berlin School of Mind and Brain, Humboldt University of BerlinBerlin, Germany; ^9^Institute of Philosophy, University of RostockRostock, Germany; ^10^Department of Psychosomatic Medicine, University Clinic of RegensburgRegensburg, Germany; ^11^Institute of Mental Health Research: Mind, Brain Imaging and Neuroethics, University of OttawaOttawa, ON, Canada; ^12^Institute of Medical Psychology, Ludwig Maximilian University of MunichMunich, Germany; ^13^Department of Theory and History of Psychology, Faculty of Behavioral and Social Sciences, University of GroningenGroningen, Netherlands; ^14^Research Centre Neurophilosophy and Ethics of Neuroscience, Ludwig Maximilian University of MunichMunich, Germany; ^15^Section of Neuropsychiatry, Department of Psychiatry and Psychotherapy, University of FreiburgFreiburg, Germany; ^16^Experimental Psychology, University Hospital of Psychiatry and Psychotherapy, University of BernBern, Switzerland

**Keywords:** brain, methodology, mind, neuroscience, psychology, systems science

## Abstract

Neuroscience is a multidisciplinary effort to understand the structures and functions of the brain and brain-mind relations. This effort results in an increasing amount of data, generated by sophisticated technologies. However, these data enhance our *descriptive knowledge*, rather than improve our *understanding* of brain functions. This is caused by methodological gaps both within and between subdisciplines constituting neuroscience, and the *atomistic approach* that limits the study of macro- and mesoscopic issues. Whole-brain measurement technologies do not resolve these issues, but rather aggravate them by the complexity problem. The present article is devoted to methodological and epistemic problems that obstruct the development of human neuroscience. We neither discuss ontological questions (e.g., the nature of the mind) nor review data, except when it is necessary to demonstrate a methodological issue. As regards *intradisciplinary* methodological problems, we concentrate on those within neurobiology (e.g., the gap between electrical and chemical approaches to neurophysiological processes) and psychology (missing theoretical concepts). As regards *interdisciplinary* problems, we suggest that core disciplines of neuroscience can be integrated using systemic concepts that also entail human-environment relations. We emphasize the necessity of a *meta-discussion* that should entail a closer cooperation with *philosophy* as a discipline of systematic reflection. The atomistic reduction should be complemented by the explicit consideration of the *embodiedness* of the brain and the *embeddedness* of humans. The discussion is aimed at the development of an explicit methodology of *integrative human neuroscience*, which will not only link different fields and levels, but also help in understanding clinical phenomena.

## Introduction: The Mosaic of the Neurosciences

Neuroscience has provided a huge amount of knowledge of the structure and function of the brain. However, an increasing number of publications makes it difficult to estimate the epistemic value of these results, their interpretations and their practical relevance. For this reason, we want to discuss some methodological problems that arise in different branches of neuroscience that may hinder integration of these branches into a unitary science. Methodology in this context does not mean merely listing presently used techniques but a theory of the epistemic value of these techniques. This goal, i.e., an analysis of the methodology of neuroscience, is concretized below in Section “The Aims of the Article”. To avoid misunderstanding, we should first say what this article is *not* about.

It is not a comprehensive review of facts and findings of neuroscience. Such a review is hardly possible within a limited volume of a journal article. Accordingly, we do not claim that our analysis is a balanced reflection of the actual trends and advances of neuroscience; particularly, it is rather brain-centered and may therefore underestimate the importance of the studies of the peripheral nervous system. Also, we do not discuss ontological aspects of neuroscience. Such questions as “Are brain and mind identical?” or “Does human “self” really exist?”, however intriguing they are, have no place in this text. The article is not about what the brain and mind are, but rather, about whether and how we (mis)understand them.

### Terminology, Epistemic Objects and Self-Understanding of Neurosciences

Neuroscience is the multidisciplinary study of those who are “working on the nervous system” (e.g., Journal of Neuroscience^1^). Neuroscience involves disciplines ranging from physics over biology to psychiatry and psychology and pretends to include sociology, economics and anthropology. This interdisciplinary character of neuroscience has a charming affordance for cooperation across nearly all academic fields. On the other hand, this plurality overshadows the serious conceptual, methodological and theoretical problems both within each participating discipline and between them. Combinations of different scientific disciplines suggest interdisciplinary linkages between different concepts, methodological strategies and contexts. Thus we find composite hybrid-disciplines such as *neurophysics* and *neurochemistry*, but also *neuroeconomics, neuroethics*, etc. However, these research areas are applications of the corresponding classical disciplines with their specific methodology.

Each component in this mosaic of the participating academic disciplines has its specific *epistemic objects, concepts, techniques, methodologies, paradigms, models* and *theories* and operates in different *contexts*. Therefore, each component approaches the common set of questions (e.g., “How does the brain work?”; “How is its work linked with our human experience?”) from a specific viewpoint. It is not yet clear how that heterogeneous “multitude of perspectives” will contribute to an integrated understanding of the brain and its functions (Chalmers, 1996; Latour, 1999; Block, 2007). What are the conceptual, methodological and epistemological requirements that neuroscience should fulfil to construct an integrated and consistent picture of mind and brain?

### Can the Subdisciplines of Neuroscience Be Integrated?

This general question can be specialized in several methodological questions:

– How close are the *views* of the different neuroscientific disciplines to each other? Are these views only *associatively* related to each other, do they *complement* or even *overlap* each other?
– Should we accept the *methodological plurality*, or can we reduce “higher level” disciplines to “lower-level” disciplines? Does the notion of “integration” (if, e.g., psychology would be integrated into neuroscience) imply that the corresponding discipline would lose its specific status and fully dissolve in the new disciplinary complex? If yes, does it hold true for all participating disciplines or only for some of them? Does the richness of the relationships between neuroscience and other sciences indicate that neuroscience is a *universal* or *unifying science?* Do concepts and methods of neuroscience replace or integrate concepts and methods of other disciplines?
– Do we have a strict methodology in neuroscience that, e.g., reminds that any localization of mental functions must demonstrate not only the *necessity* of the respective structure for a specific function but also its *sufficiency* for this function?
– Do we have sufficient *theories* already? Do mathematical models capture mental phenomena?

Presently, we do not claim to answer these great methodological questions. We also do not propose any specific perspective, although we have a clear preference for a systemic and even ecological extension of the view of neuroscience; but this view should be worked out later in a special initiative.

### The Aims of the Article

Regarding these basic questions, in the following sections we shall discuss six main points:

*First*, we shall give an overview on common *taxonomies of science* to delineate borders between the disciplines participating in neuroscience.
*Second*, we shall elucidate specific methodological problems existing within *neurobiology* as a natural science and how these problems complicate the attempts to relate neurobiology to mental functions.
*Third*, we shall explore current intra-disciplinary problems of *psychology* because any studies of the neural basis of mental functions cannot be better than the concepts, methods and theories of psychology that are used there.
*Fourth*, we shall analyze principal methodological difficulties in the studies of *mind-brain relations*; these methodological issues can carefully be put apart from the metaphysical aspects of the mind/brain problem.
*Fifth*, we shall summarize the advances of integrating modern *philosophy* with its various subdisciplines into the ensemble of neuroscientific disciplines.
*Finally*, we shall describe the prospective utility of *Systems Science* as a potentially powerful integrator of neurosciences.

## The Structure of the Field of Neurosciences

### Summary

An essential *methodological pluralism* of neuroscience appears necessary independently of the ontological position of a researcher (i.e., monism, dualism, or pluralism). Regarding methodological reductionism, we do not rule out the possibility to reduce some aspects of cognitions and emotions (e.g., impaired working memory) to their neural basis (e.g., deficient prefrontal networks: Schaffner, 2013), but such partial reductions do not yet prove the possibility to reduce the whole. Furthermore, integration cannot be regarded as a simple coexistence of concepts from different subdisciplines, but presumes a considerable overlap of these concepts.

### Some Preliminary Distinctions

Structuring the field of sciences is a subject of the *history of science* (Hacking, 2002) and the *philosophy of science* (Bunge, 1998). One basic dichotomy is that between “factual” (empirical) and “structural” (theoretical) sciences (Bunge, 1998). Examples of the latter are cybernetics, mathematics and systems science. More detailed analyses of similarities and differences between sciences and their practical applications are often used to construct taxonomies of sciences (Bunge, 1998). A scientific discipline—for instance, psychology or neurobiology—can be characterized by its *epistemic object* (mind/brain), its *concepts* (consciousness/discharge pattern), its *methods* (questionnaires/EEG), its quality of *data scales* (ordinal behavioral data/rational voltage changes), by *specific phenomena* (anxiety/neuronal activity) and its *theories* (frustration theory of aggression/Hodgkin-Huxley-equation). Different disciplines also frequently have different implicit ontological and metaphysical assumptions. Neuroscience as an epistemic program is based on a very heterogeneous set of concepts, methods, paradigms and theories. Successful integration would imply the need to define each participating scientific discipline in order for them to be able to support each other.

First, regarding the *epistemic objects* of scientific investigation the core disciplines of neuroscience study electrically active molecular structures (e.g., ion channels; *physics*), gene expression (*genetics*), synaptic processing (*molecular biology, pharmacology*), growth and wiring of neurons (*histology*), structure of brain areas (*anatomy*), or plasticity of circuits (*physiology*). The great challenge for neuroscience is related to neural mechanisms of “higher” or “mental” information processing (*psychology*). Disciplines like psychology can also work without referring to a material substrate like the nervous system.

Second, regarding the *concepts*, some of them (like *discharge* or *BOLD signal*) are technology-based observational terms emerging from physical measurements, while others (like *consciousness* or *sensation*) are subject-related observational categories.

Third, regarding the *methods*, there exist sophisticated *physico-chemical measurement technologies* in the present neurobiological laboratory. The opposite pole is presented by data that rely on *verbal reports* specific for psychology or sociology. Somewhere in the middle are methods of direct observation of behavior, which can be of different level of complexity and result in data varying from purely qualitative observations to exact records that allow strict mathematical analyses, e.g., in kinesiology. Techniques alone, which can be learned within a few months, are not sufficient for the appropriate interpretation of results without the *theoretical context* underlying these techniques. A psychologist can record single cell activity and a physicist can perform an experiment in social psychology, but this does not yet mean that they really know how to conceive of the data they obtained.

Fourth, the quality of *data* can be used to distinguish “hard” sciences like physics with *interval or rational data* from “soft” sciences such as sociology that is based to a large amount on *qualitative or ordinal data*. This implicates a proper choice of statistical tools for data analysis.

Fifth, *discipline-specific phenomena* that drive specific research can be identified. On the one hand, some of these phenomena have originally come from philosophy (the self) or folk psychology (attention). On the other hand, purely physical (e.g., electrical) phenomena can also belong to the domain of neuroscience.

Sixth, *theories* can be expressed on a highly elaborated *formal level*, or only formulated on a *verbal level*.

Reviewing these criteria, the roughest *dual classification* of the (empirical) sciences distinguishes between natural and social sciences (“humanities”; e.g., Dilthey, 1907/1954; Von Wright, 1971). Many authors further distinguish between “mind sciences” and “social sciences” properly, which leads to a trichotomy rather than dichotomy. The argument runs that the social domain emerges as a relatively autonomous system that cannot be reduced to single persons or even groups or populations of humans (e.g., Luhmann, 1994; Mayntz, 2006). Social issues, according to Durkheim (1895/1982), are not merely the presence of other persons but real or virtual interpersonal relations like expectations of behavior or expectations of expectations. If social processes cannot be reduced to individual behavior, they can be related but not reduced to individual neurobiology.

A resulting tripartition of the sciences reminds of the “three worlds” of Popper (1972) who distinguished a “world 3” of cultural artifacts from the mental “world 2” and physical “world 1”. Similar three-world conceptions were also suggested by other philosophers (Frege, 1918; Carnap, 1967/2003).

One can argue, however, that important differences exist not only between the mental world and the social world, but also between the social world and the cultural world. Therefore, more and more fine-graded classifications are possible.

### Boundaries Between the Neurosciences

Already at the first glance the number of differences between the various disciplines makes it inappropriate to include purely psychological studies into “neuroscience” because the data of such studies only indirectly indicate processes in the participants’ brain. Therefore, we prefer a narrow definition: a study belongs to neuroscience only if it also uses *physical or chemical measures* of the structure and the actions of the nervous system. A neuroscientist is a person who is registering, analyzing, manipulating or modeling a physico-chemical property of the nervous system.

#### A Dual Structure of the Neurosciences

The simplest classification of sciences (i.e., natural vs. social/cultural sciences) would roughly correspond to a dichotomy between the *basic* neurosciences, which studies the “isolated” brain, and another field of *behavioral, affective and cognitive neuroscience* (BACN), which studies aspects of the brain as a part of the organism-environment system. The former is a purely natural science and investigates structures and processes in the nervous system using methods of natural sciences. The latter looks for relationships between these physiological processes and a different class of processes called “psychological”. This division between the basic neuroscience and BACN can be criticized and overcome in a further discussion but it is necessary as a starting and branching point.

Such a dichotomy roots, first of all, in the history, because neuroscience developed mainly from neuroanatomy and neurophysiology. This essentially biological approach remains the basis of all other (cognitive, social, ethical, etc.) neurosciences also in the sense that any methodological *flaw* at this basic level necessarily leads to misconceptions at all other (“upper”) levels. The neuroscience as a purely natural science completely relies on conceptualizations of experimental or theoretically-derived data based on the *third person* perspective. In contrast, BACN has to take into account also the *first-person* perspective.

This distinction is also important because it is precisely BACN that is the object of great social debates. The public attention is bound not to details of electrical and chemical processes occurring in neurons and glial cells, but to how these processes are related to our feelings, decisions and (social) behaviors. Thus we have to consider the methodological issues of BACN, as it entails steady comparison between the data obtained using the methods of natural sciences, on the one hand, and those obtained using the methods of “humanities”, on the other hand.

#### Social Neuroscience—The Neurobiology of the Second Person Perspective?

If social processes cannot be reduced to the processes “under the skin” of a single human, then social neuroscience also cannot be reduced to BACN. Therefore, social neuroscience may be regarded as a third field of neuroscience (Society for Social Neuroscience, 2014).

Social behavior of humans obviously includes general psychological components such as perception, cognition, memory, etc. However, the question is whether the corresponding neuronal brain networks involved in processing *social* stimuli are different from the networks involved in these general mental processes. An example of social neuroscience might be, e.g., a study of how the brain is working when a subject is involved in political affairs. This type of studies is closely related to the quest for a “cultural neuroscience” (Han et al., 2013).

Another frequently forgotten aspect is that the social component interferes in each experiment in human neuroscience: whenever an experimenter tries to obtain the informed consent from a human subject and discusses the conditions of the experiment with the subject, this is already a social process that can considerably affect experimental results.

#### Interdisciplinarity, Reduction or Integration of Methods?

We tried to show that neurosciences could rely on several methodologically different disciplines. In this relation, Northoff (2014a,b) speaks about our structural and functional brain our mental brain and our sociocultural brain. When, however, an integrated picture of the brain and its functions is aimed, three different strategies may be used.

##### Reduction

The most respectable tradition in neuroscience is to dare the great challenge of *reduction* of all this disciplinary complexity to neurobiology. This is not the only tradition. Many brilliant neuroscientists rejected the idea of reduction, including Gustav Fritsch, Sir Charles Sherrington, Sir Edgar Adrian, Sir John Eccles, Wilder Penfield, Ragnar Granit and many others. But the reductionist stance plays nowadays a particularly strong part. As stated above, the *core of neuroscience* is a natural science of *neurobiology*. If, however, neuroscience claims to explore mental and social events, it has to employ knowledge and methods going beyond its biological core, i.e., those of psychology, sociology and perhaps other disciplines. To be successful, such a reduction has to fulfil at least two criteria, i.e., to replace mental concepts with neurophysiological concepts (conceptual reduction) and to discover bridge laws that would permit to deduce mental processes from physiological processes (theoretical reduction; Nagel, 1961; Rosen, 1991).

At least presently, there is no sign of how the two above criteria could be fulfilled^2^. Therefore, the methodology of neuroscience is necessarily pluralistic, or at least dualistic. Note that this methodological pluralism does not mean a metaphysical pluralism or dualism, but can well agree with a metaphysical monism. We can believe that “at the very end” pain and activation in the pain matrix of the brain are one and the same thing, but in a particular neuroscientific study, we necessarily use *two* different approaches to pain, such as subjective reports and direct brain measures.

##### Pluralistic methodology

An alternative to reductionism can be the “interdisciplinarity” of neuroscience, easily understood in the anarchistic sense of “non-disciplinarity” or even “antidisciplinarity” (Feyerabend, 1975). We think, to the contrary, that the real interdisciplinary collaboration requires clear awareness of, and a critical attitude to, the limits of any discipline that is given by its traditional subject, current concepts, methods and theories. Using a microscope is not sociology and using a clinical interview is not biology. Additionally, the methodological integration of practical applications of neuroscience (e.g., in medicine) is an important aspect that will permit us to go beyond the mere “interdisciplinarity” into the domain of that has been called “transdisplinarity” (Mittelstrass, 2011). Transciplinarity is characterized by the equipotent *interaction* between *researchers* and *practitioners*.

##### Perspectives of an integrative neuroscience

The third, integrative approach to neuroscience is opposed to both reductionism and unstructured pluralism. However, what does “integrative” mean? Any attempt to develop an integrative neuroscience has to deal with *epistemic compatibility* of the various methods. The usual solely additive combination of methods and levels may not be sufficient to construct a comprehensive picture of neuropsychological phenomena. Only theoretical efforts seem to promise integration by building conceptual bridges. Integration is not a juxtaposition of concepts, but rather their overlap.

An open question remains, however: does integration warrant identity of the component disciplines or lead to resolving the borders and fusing the disciplines? This issue should be discussed intensively in the context of methodology of neuroscience.

Such semantic aspects are only vaguely depicted by stakeholders of the program of an integrative neuroscience. Some examples (without a claim for completeness) are: (i) journals aiming at a synthesis of the results of brain research for understanding complex behavior (Frontiers in Integrative Neuroscience, 2015) or at the integration across “hierarchical levels” (World Scientific Journal of Integrative Neuroscience, 2015); (ii) research institutions targeting integrative multi-level research including humanities (University of Tübingen, Centre for Integrative Neuroscience, 2015); (iii) educational programs also aiming at the integration of perspectives including anthropology, philosophy, history (Binghampton University)^3^ and computational science (Fordham University)^4^.

Unfortunately, problems of integration do not start only when we are trying to connect several disciplinary traditions. Rather, particular disciplines have their own internal methodological gaps that should be minded when they contact each other.

## Neurobiology—Some Intradisciplinary Methodological Difficulties

### Summary

There are considerable internal methodological problems within the purely neurophysiological division of neuroscience. Neither plenty of data, nor the diversity of techniques can yield profound understanding without general principles of brain function. These problems can undermine the whole project of neurobiological explanation of mind. A misconception regarding neurophysiological basis of a brain phenomenon results in a high probability of wrong hypotheses about how this phenomenon is related to mind and behavior.

### Experimental Neurobiology: Do More Data Implicate More Understanding?

Empirical research in neuroscience is largely technology-driven: imaging studies, electrical and magnetic stimulation and recording methods, microarrays, optogenetics, etc. are rapidly developing technologies that generate more and more data. We have now unprecedented access to the complex spatial-temporal patterns of electrical activity and biochemical processes accompanying specific cognitive or behavioral states.

Such large-scale and multi-site recordings of spatial-temporal electrochemical patterns may fill an important gap in empirical brain research, namely the hiatus between molecular-cellular and systems neuroscience. However, there are still serious challenges, such as the enormous diversity and complexity of neurons, beginning with their multimodal classification systems involving electrophysiology, cytology, histology, biochemistry and molecular biology. It turns out extremely difficult to unravel neuronal connectivity and signaling even in relatively simple model systems. An unanswered question is how far we may generalize from less complete data and experimental models. How much diversity must be considered to explain brain phenomena, and how much can we rely on general principles?

Unfortunately, errors at this level can undermine the whole project of neurobiological explanation of mind. Thus a few decades ago many scientists claimed that voluntary choice is an illusion after Libet (1985) showed that readiness potential (RP) appears long before the subjects make their voluntary choice. Now the falsity of this conclusion became obvious as we learned that RP, related to the activity of the premotor cortex, cannot be responsible for a choice of some particular movement (e.g., Herrmann et al., 2008). A fissure in the basement of a building makes unstable all floors that are built upon it.

Heterogeneous data, ever increasing in amount, are analyzed by sophisticated multivariate techniques. As a result, data mining substitutes theory-driven considerations in several fields of neuroscience. Large amounts of data enrich our *descriptive knowledge* but not necessarily imply high *explanatory power*. The explanatory power depends on the strength of *principles* that have high heuristic potential. Explanation is an account that allows to subsume a new observation to a general regularity: the force of a crashing car can be described, explained and predicted by the laws of motion by Newton, if the mass and the acceleration of the car are known. No comparable general principles that explain mental events and their neural basis are known in brain research (Craver, 2007). Some suggest that a mechanistic explanation is sufficient if a micro-mechanism can explain a macro-phenomenon. However, this becomes increasingly difficult when moving towards higher-level functions. Already the understanding of neuronal network synchronization engages hundreds of experimentalists and modelers, many of whom do not start with such basics as ion channel activity but rather at the level of neuronal spike trains. Explaining the interactions between different brain areas on the basis of ion channels will be an even more difficult task.

The most extreme explanatory power of principles derived from experimental research would be a complete deterministic explanation of the brain, as exemplified in Laplace’s famous “demon”, that was suggested to predict everything in the future on the basis of the knowledge on the position and the velocity of each single particle in the present. Beyond possessing this universal set of data, the demon has, of course, to know all basic physical laws. The necessity of this additional, i.e., theoretical, knowledge was so obvious for Laplace that he even did not worry to mention it explicitly.

However, there is no law in neuroscience whose status might be compared with that of Newton’s laws. Therefore, even if Laplace is right and even if his demon knows every single atom and ion in the brain, it would not be able to predict future behavior, because it does not know those general laws that determine the dynamics of the system.

### Different Methods—Different or Integrated Pictures of the Brain?

The methodological diversity of modern neurobiology is a very positive development because each method possesses its own advantages and disadvantages and, thus, different methods can successfully complement each other. However, the belief that “more different methods” would automatically yield deeper understanding of how the brain works is not much better than the belief that “more data” would yield such deeper understanding.

Already the conceptual link between chemical and electrical signaling in synaptic transmission is not sufficiently understood: the synapses with their multiple feedback loops (Abbott and Regehr, 2004) are not switches or transformers but adaptive filters, forming highly complex micro-processors. Further, the spatial and the temporal domain are not yet linked directly to each other: fMRI has a good spatial resolution, EEG has good temporal resolution, and the relationship between both signals is still subject to research, despite considerable progress. In the 1990s the technical problem of how to combine the two was solved, but notably, this technical progress has not been accompanied by a corresponding progress in scientific knowledge. Simultaneous EEG/fMRI recording is still rarely performed, and it has not yet resulted in any major breakthrough. Thus beyond technical opportunities, theoretical and conceptual ideas are necessary. To usefully perform a combined event-related potential (ERP)/fMRI registration, we should ask the question: which specific theory of brain function could be tested by this registration (Platt, 1964)?

Despite these challenges of methodological integration, combined recordings with different technical methods addressing the relation between macro-level and micro-level are of eminent importance, especially the correlation of fMRI data with single unit activity (Logothetis, 2008). However, the fundamental question of whether a combination of technologies can close the epistemic gaps between different system levels remains open.

### Data Analysis and Modeling

Most techniques of data analysis imply a sort of separation between “signal” and “noise”. This separation process frequently involves spatial and temporal averaging, filtering, smoothing, or normalization, i.e., reduction to a Gaussian form. Doing so, we additionally reduce the set of data which is already extremely small as compared with what is really going on in the brain at that time. In the basis of these procedures lies the idea formulated by William James that “The art of being wise is the art of knowing what to overlook”. A lot of data is overlooked when we extract their “substantial” part and ignore the “less important” one.

The exact meaning of the notions “signal” and “noise” is, however, not always clear. Often the signal is defined as the activity pattern that remains stable with stimulus repetition, whereas noise is everything which changes from one stimulus presentation to another one. However, sometimes it is exactly the change which is of most interest, e.g., in case of habituation, fatigue and plasticity. This limitation is particularly serious if we compare such data with the contents of consciousness, while knowing that consciousness is rather a stream than a state. The poor temporal resolution of many sophisticated techniques we use for study of the human brain further aggravates this problem.

Another definition of signal is the exact time-locking of the brain activation to a stimulus presented by an experimenter. All the remaining activity, which is not time- and phase-locked to this stimulus, is declared “noise”. Kotchoubey (2006) characterized this view as a “mania grandiosa” of the experimenter who believes that the human brain, bombarded by thousands of stimuli every moment, is particularly busy with that one stimulus that he (the experimenter) is interested in. In fact, phase-locked and non-phase-locked, as well as pre-and post-stimulus EEG effects relate to each other, and processes starting as anticipatory activations prior to stimulus presentation may continue after this presentation modifying so-called “responses” to stimuli (Fingelkurts et al., 2002, 2003; Kotchoubey, 2006). When our attempts to concentrate only on the useful portion of the brain signal are combined with the lack of an adequate definition of “usefulness”, terabytes of information already available are lost.

Ways to overcome these limitations are sought for in data-driven modeling that has become an important approach in many fields of experimental neurobiology. This approach starts with the choice of the appropriate mathematical method that can reduce complexity of the data, especially those coming from new high throughput technologies such as microarrays. Therefore, tools from multivariate statistics are used (principal component analysis, cluster analysis, independent component analysis, support vector machines, etc.).

By these procedures, for instance, machine-based algorithms can be constructed that allow to classify data patterns into “pathologic” or “normal” categories. As humans have difficulties with comprehending data representations by visual displays that extend more than 5–7 variables, machine-based algorithms are unavoidable. At present, the methodological commitment is devoted to exploring “big data” sets with sophisticated mathematical tools that allow for the identification of clusters that help to predict behavior patterns. Neuroscience continues this development largely starting in molecular systems biology with its strong roots in mathematics (Kitano, 2002; Le Novère, 2007; Tretter et al., 2010).

### Animal Experiments—How Validly Do They Represent Human Mental States?

Animal experiments are unavoidable in brain research. Our knowledge on the mechanisms underlying some neurological diseases (such as epilepsy) is mostly based on such experiments. Regarding psychiatric diseases, however, the development and choice of appropriate animal models is still an issue of debate (Koch, 2006). There is no appropriate animal model of shame, guilt, or paranoia; however, these phenomena are of huge importance in mental diseases. Animal models based on behaviorist epistemology, and even highly elaborated experimental designs (e.g., inducing depressive behavior by deprivation from mother animals) cannot capture significant pathological features (e.g., feeling listless and committing suicide).

## Psychology: Behavior and Subjectivity

### Summary

The aim of human neuroscience is frequently regarded as a search for “neural underpinnings of” mental functions. This understanding presumes that mind sciences (mainly psychology, but also psychiatry and psychopathology) already have at least an approximate list of the functions whose underpinnings we have to investigate. However, presently there is no such list. There is a steady tension between the uncritical use of everyday words, on the one hand, and a loss of any link between the operational definitions of a term and its phenomenality, on the other hand. At the same time, a new conceptual framework arises that may be regarded as a first step to integration of clinical phenomenology, neurobiology and computational psychology.

Another conflict within the present-day psychology is between first-person and third-person perspectives. Any reduction of one of them to the other one seems inappropriate. Rather, a process of integration can profit from the acceptance of methodological dualism between behaviorist/neurobiological and experiential/phenomenological positions. From a purist’s standpoint, this may appear awkward and can eventually produce category errors. From an eclecticist’s standpoint, however, this status reflects an unsolved mind-body debate in a warranted position of “live-and-let-live”.

### Objects of Psychology and Duality of Methodology

If psychology is defined as the science of *experience and behavior* (Zimbardo and Johnson, 2011), it has two different kinds of epistemic objects: on the one hand, first-person phenomena that apparently do not have precise spatial localization and extension, on the other hand, observable behavioral changes that can be identified exactly in space and time. This juxtaposition of phenomenal concepts (experience) and natural science concepts (overt behavior) justifies a methodological dualism. Well-known examples are a Spartan who can have pain without showing it and a simulant who can express emotions but doesn’t have it.

An attempt to base psychology on a purely objective methodology and to avoid the methodological dualism was undertaken in the middle of the 20th century under the name of *behaviorism*: the mind science should be reduced to space- and time-related processes like those in any natural science, i.e., the objectively observable behavior. Behaviorist psychology was suggested as a scientific alternative to that subjective psychology based on introspection, which was discounted by reductionist philosophers as merely “folk psychology”. Watson (1919) and Skinner (1974) proposed their behaviorism as the only appropriate approach for scientific psychology. This view intended to eliminate all subjective concepts, and especially intentional concepts, by observational concepts. The subjective dimensions of mind should be eliminated. In accordance with this view, *behavioral neuroscience* does not have the problem of the dual methodology because both neurophysiological and behavioral processes can be described in the same third-person way.

However, radical behaviorism did not succeed. Already in the 1930s it became clear that intervening variables cannot be removed from psychological experiments. Conceptions of behavioral experimentation proved especially insufficient for clinical psychology and other fields of applied psychology. For these and other reasons, the so-called “cognitive turn” in the 1960s resulted in an encompassing rejection of the rigid behaviorist restrictions.

Presently, numerous psychologists and neuroscientists claim to investigate subjective aspects of mind. However, in many applications the rests of behaviorist thinking are still at work. Many leading psychiatrists try to base the diagnostics of mental disorders purely on patients’ observable behaviors, ignoring their subjective experience. These attempts are not in line with the practical clinical work that shows that patients tell us what they are experiencing (Parnas, 2014). For instance, the use of recreational drugs could be conceived of in terms of “reward”, but this concept ignores the variety of drug users’ motivations, e.g., to make new experience or to explore one’s subjective position in the world. The term “reward” is too general to be of high explanatory value.

The level of *meanings*, which is the core of subjectivity and phenomenology, cannot be eliminated from psychological science, yet may even have priority building a semantic basis for objectivation and operationalization of concepts and constructs (Harvard Law School, 2015). This means that neuroscience also cannot avoid the methodological dualism if it wants to attain its declared goals.

### Operationalization of Psychological Constructs

In line with these methodological accounts, it is important to note that most psychological termini, from pain to falling in love, have their roots in subjective experience and folk psychology. When a lay person asks what science (psychology or neuroscience) can tell us about these phenomena, she is interested in the meaning these terms have in the ordinary language. Therefore, subjective experience is the starting point of most psychological investigations. In consequence, in experimental psychology (a fortiori neuroscience), these phenomenological constructs have to be operationalized as measurable, controllable variables. For instance, emotions are commonly operationalized as a triad of elements: (a) a specific feeling and the ensuing cognitive appraisal; (b) a specific (observable) emotional behavior; and (c) a specific physiological response. Thus experiential descriptors are combined with biological descriptors. Regarding this methodological plurality, it seems to be fruitful if phenomenological constructs have to be broken down to testable operationalization as it is the case in the attempts to localize the phenomenological self in the brain: self-related processing is a testable setup that helps to approach the brain structures that are at least necessary for self-perception of a person (e.g., Northoff, 2014a,b).

However, when scientists re-define the terms of subjective experience to make them easier for experiments, they should take in mind that a result of this operation can be the terms having lost the meaning they have for most people (including those who fund the studies). For example, psychologists can define their aim as a study of aggression, but in their real experiments participants merely observe schematic pictures of violence. Even though the results can be meaningful, they may not give a new insight into the domain of aggression as this word is understood by the broad public. Moreover, such operational definitions of mental phenomena, though simplifying our experimental work, make an impression that the object of the study (e.g., “love”) is a single mental process, which is not at all evident. Love is an object-related multi-level process entailing cognition, memory, expectation, emotion, motivation and possibly other components.

The said concerns not only experimental, but also clinical psychology and psychiatry. As Andreasen (2007) pointed out, some psychiatric operationalizations like rating scales for mental symptoms may lead to a loss of the phenomenon. Can a system of concepts be built that allow empirical operationalization, in the same time preserving links to real experience?

### Basic Concepts—Listed but Not yet Nested in a Network

Psychology textbooks describe several main categories like perception, thinking, emotions, etc., each of which is subdivided into subcategories. Together, this means 30–50 significant psychological categories, whose interrelationships are often unclear. This indicates the need for an integrated *taxonomy* of mental states and processes that can be constructed on the basis of observational data (e.g., Fodor, 1983; Tanida and Pöppel, 2006).

A well-known functional model taxonomy postulates that a system entails several elements: a *sensor* that receives the stimuli, an* effector* that generates the behavior, a *processor* that analyses and synthesizes information, a *depositor* whose function is the storage of information, and an *evaluator* to declare the relevance of the results of the processor. As this cascade of operators is related with the environment a *functional circle* (von Uexküll, 1920) can be constituted, which also includes planning (control loop paradigm) as well as plan-related and memory-based *expectations* regarding the effects of behavior. Several other, more segmented taxonomies exist as they are constructed mainly in computational cognitive science (Sun and Franklin, 2007).

Intrinsic dynamics of psychological processes can be illustrated by an analysis of the category “emotions”. Thus some emotional states are opposing each other (e.g., Plutchik, 1991; Ekman, 1992). Such antagonisms were specified in the *opponent process theory* of emotion and motivation, which states that an adaptive slow down-regulation of the level of neutral emotions takes place (Solomon, 1980). A similar functional opposition exists in the domain of color perception (e.g., a red afterimage after seeing green). This idea of opposing forces corresponds well with neurobiological findings suggesting simultaneous and successive inhibition or opposing interplays between nucleus accumbens as a reward-related structure and the amygdala as an anxiety-related structure. Similar taxonomies of emotions have been developed in neuroscience (e.g., Panksepp, 1998) and philosophy (Ben-Ze’ev, 2001).

The above concepts mainly originated from cognitive science. On the other hand, ecological psychology (Gibson, 1966, 1979; Stoffregen, 2000) claims to propose its own, “organism-centered” conceptual system, which is sometimes regarded as a strict alternative to the network of cognitive concepts. Although the ecological view is much less developed, it has several advantages, particularly a strong anti-atomistic attitude and an accent on the close-loop relation between perception and action (in contrast to the view on the action as a response to perceptual events). Ecological psychologists view perception not as mere processing of sensory information but as a set of intrasensory actions (e.g., touch, eye movements) aimed as an active search for relevant information (e.g., Turvey, 1996; Noe, 2005). We believe that the presumed opposition between the cognitive and ecological approaches has mainly historical reasons, and that the two approaches are interrelated like Descartes’ axial coordinates and polar coordinates, and that a future taxonomy of psychological concepts will include a set of translation rules between the two.

Moreover, some branches of ecological psychology work in close relationship with physiology of movement (e.g., Thelen, 1995; Turvey, 1996). Behavioral acts, the main observable of psychology, are, from a physical point of view, movements of the whole body or its parts. Nevertheless, psychology and movement science have been developed largely independently of each other (Kotchoubey, 2012). From the methodological viewpoint, psychology suffers even more from this separation because the modern kinesiology is a strict, highly mathematized science with a well elaborated, though limited, conceptual apparat and exact experimentation (Latash, 2008).

Another contemporary development of ecological ideas is the studies of gene-environment interaction (review by Manuck and McGaffery, 2014). These studies can be conceived of as realization and specification of the concept of “environment” as a subject-related term (“Lebensraum”: Lewin, 1939) as contrasted to the definition of environment in objectivistic physical terms proposed by the founder of ecology (Haeckel, 1899/1992).

We suggest that the difference between the aspects of environment is closely related to the general psychological tension between the first- and third-person perspectives. We also believe that constructive application of the systems approach has a potential to bridge both presently existed methodological gaps (between “objectivist” and ecological psychology, and between behavioral and movement science). We shall return to this issue in Chapter “Systems Science” below.

### Process Models—Towards Integrative Systemic Computational Models

One of the most stimulating current concepts is the predictive coding model that conceives of human information processing as determined by the processing of expected (or predicted) vs. observed stimuli: activation occurs only if an *observation is unexpected* (Sokolov, 1963; Grossberg, 1982). However, if an expected reward does not occur the behavior is suppressed (Dayan and Berridge, 2014). The model presumes not only that experience-based observations are connected with output elements but also that an action plan is connected with the observed result by a feedback loop. In this view, *reward* can be seen as the result of a *prediction error* (Schultz, 1998, 2013; Niv et al., 2012). This network-related concept of reward (Friston, 2012) further stimulated the development of the fields of “computational psychology” and “computational psychiatry” (e.g., Montague et al., 2012). It is related to the neurobiological concept of “corollary discharge” (Sperry, 1950), which has recently been applied to theoretical understanding and measurement of altered self-experience in schizophrenia (Mathalon and Ford, 2008; Rösler et al., 2015). In this case we see a fruitful integration of several conceptual fields from behavioral biology to clinical psychology and psychopathology, linked by neurobiologically based systemic conceptual models (see Chapter “Systems Science” below).

Some further examples indicate new roadmaps to a *systemic psychology* (and psychopathology):

– *Decisions* can be regarded as the result of internal cycles between cognitive and emotional processes (Heckhausen and Heckhausen, 2010). This qualitative model is also framed by the concept of a global feedback loop between action and its consequences that result in further modifications of action plans (Miller et al., 1960).
– *Binding behavior* can be seen as the result of the feeling of dependence minus the feeling of security (Bischof, 1975). Therefore, if the latter is strong, the binding behavior is reduced even with a high level of dependence. Each of these states and drives is based on other elementary mental states and processes. For instance, the feeling of security is the result of the experience of familiarity and closeness.

To illustrate self-organized state trajectories, even complex constructs such as *the self* can be conceptualized and modeled in the context of systems theory as an attractor (i.e., a certain processual structure) at certain time scales and in the presence of conditions that may calibrate or destabilize the attractive self-structure (Tschacher and Rössler, 1996; Tschacher and Haken, 2007; Tschacher and Munt, 2013). In this way, issues of experience can be conceptualized and modeled in a way that allows operationalization and categorization of its dynamics.

## Methodological Issues of Brain-Mind Studies

### Summary

Models of one-to-one correspondence between a mental process and a morphological or biochemical structure can successfully work in selected cases; however, even in such cases further development reveals that these models are only approximations whose productivity is limited. Understanding a mental function as translated into a temporal structure of ons and offs of mental activities and related to a network model is much more complex but, as the working memory paradigm indicates, not unrealistic.

Thus a new neurophysiological or neurochemical finding always implies a *functional interpretation* on the psychological level, and the history shows that sometimes such interpretations are arbitrarily isolated and oversimplified. To avoid this, a kind of a “neuropsychological uncertainty relation” can be suggested: the more precise is location in the brain, the less exact is the (psychological) function, and vice versa: the better can we define the function, the more difficult is its localization.

### Levels of Correspondence

Brain-mind relations are usually measured by contingencies between altered brain variables and altered mind variables. Several distinct levels of these contingencies have to be considered.

*Correlates* are simple temporal coincidences between the two. The amygdala is activated when we experience fear; the activity of the hippocampus increases during memorization tasks; attended stimuli elicit a specific EEG wave lacking in response to non-attended stimuli. The overwhelming majority of all phenomena obtained by BACN are neurophysiological correlates of behavioral, cognitive or emotional states or processes. Some authors assume that a strong correlation between brain activity and mental functions indicate that the neuronal activity underlies these functions. Such assumption is a *metaphysical* one and cannot be justified on the basis of the correlational data only. There are data indicating that social-cultural environment can substantially affect not only brain function but even brain morphology (e.g., Paulesu et al., 2000; Bufill and Carbonell, 2004; Petersson et al., 2007). The assumption, that the brain causes behavior and not vice versa, is not justified by the mere strength of the correlation.

*Necessary conditions* are a higher level of correspondence that just correlates. For example, synchronous activity of thalamo-cortical loops does not simply correlate with conscious experience but, rather, no conscious experience is even possible (at least in adults) whenever these loops do not function properly. The necessary conditions may be more or less specific. Finally, even the basic physiological functions such as respiration and circulation are also necessary for higher cognitive functions. Other physiological phenomena can be found, however, which are more specific conditions for some functions. The most specific conditions are necessary *and sufficient*. In this case we should not only know (like in the consciousness example above) that whenever a physiological process is absent, a mental process is absent as well, but also that whenever this physiological process is present, the mental process is also present.

The *identity relation* is the highest level of correspondence. Even if a physiological process is both necessary and sufficient for a mental process, this does not logically mean that the two are one and the same process. There are several specific criteria of identity that are extensively discussed in the philosophical literature.

Our ability to capture these four levels of the correspondence between brain and mind (or behavior) strongly depends on the design of a neuroscientific experiment. Most experiments have psychological or behavioral independent variables, and neurophysiological dependent variables. Examples are given above: the activity of the amygdala is recorded as a function of the emotional state, and the electrocortical responses as a function of the state of attention. Such experimental design principally allows the researcher to obtain only physiological *correlates* of mental activity. If correlations between local structural activities and mental functions are interpreted as causal relations, this is a philosophical and not a scientific claim.

The opposite arrangement is used in lesion studies, stimulation studies (including psychopharmacology), biofeedback studies, or sleep studies. Here, neurophysiological variables are independent factors, and mental and behavioral changes are dependent variables. This kind of experiment can reveal necessary physiological conditions of a behavior or a mental function.

A combination of the two approaches is supposed to give rise to identification of sufficient conditions. To our best knowledge, necessary and sufficient neurophysiological conditions have been found out only for simplest forms of learning in lower animals (Linden, 2003). For mental processes such as subjective sensation, feeling, etc., only necessary conditions could be identified to date. Although many neuroscientists, including many contributors of the present text, believe in the principal identity of mental and physical processes, we insist that this remains a matter of belief and not of scientific facts.

### From Structure to Function

Certain brain areas critically contribute to the generation of an organismic function. An interesting example is Parkinson disease with its reduction of motor dysfunction patterns to a degeneration of dopamine cells in the brain stem (Davie, 2008). The role of this degeneration was first indicated by post-mortem neuropathology and then supported by means of the experimental induction of a Parkinson syndrome by reserpine, and by the positive effect of dopamine transmission agonists such as L-DOPA (Carlsson, 2006).

Parkinsonism, in which the impairment of a local brain structure and a local biochemistry causes a clinical neurological phenomenon, became a paradigm for successful clinical-neurophysiological reductionism. However, further research in Parkinson disease showed that a focal view is insufficient since also glutamate system participates in the development of Parkinsonian symptoms.

Apparently, simple concepts of locality of functions and single molecular players had to be abandoned since lesion studies showed for nearly every area of interest that a partial restitution of function can be attained after a specific training (Pöppel et al., 1973; Sahraie et al., 2010). Also imaging studies revealed that most brain areas are involved in multiple mental functions, and that each single mental function is related to several brain areas. For instance, already in the early 1990s Felleman and Van Essen (1991) identified more than 30 areas involved in visual perception. A network conception of representation of mental functions in the brain appears more appropriate, as was suggested by Lashley (1950) more than 60 years ago.

Above we already mentioned the problem which arises when very weak laboratory analogs of complex phenomena such as love are used (e.g., pictures of a beloved person), and the results are generalized onto the phenomenon of love in general. This problem further aggravates in neurophysiological (e.g., fMRI) studies that frequently use even simpler and more schematic stimuli, and the activated brain areas are then interpreted as “representing” or “involved in” love. An example can be Ekman pictures of facial expressions of emotions as a basis of “affective neuroscience” (Paul Ekman Group, 2015). Indeed, these pictures proved to be a powerful tool for the analysis of emotional *expression*. This does not mean, however, that they can be used with equal success in the study of emotional *experience*.

This implies that neuropsychology cannot be better than the psychology it is realized upon. When a psychological concept is weak, its relation to underlying neural substrate cannot be strong.

### From Molecules to Mind

The problem of relating structure to function remains also at the molecular level: a one-to-one relation between a molecule and a mental function does not hold true. In the past, certain neurotransmitters were wrongly classified as hormones “for” particular mental states and processes. For instance, dopamine has made a path from a pleasure molecule to an addiction molecule to a reward prediction error molecule (Schultz, 1998). Oxytocin is often depicted as a hormone of love, binding and even happiness. However, other substances such as serotonin and dopamine are also involved in binding. Binding behavior is a result of a complex set of external and internal conditions (Bischof, 1975). On the other hand, oxytocin is involved in several organismic functions such as prolactin secretion. Recent studies indicate a possible publication bias, in that the studies that do not reveal a link between oxytocin and social behavior are not published or even not submitted (Lane et al., 2016). To summarize, the biological specificity and selectivity of oxytocin are questionable. Relating one type of molecules only to a simple concept of folk psychology cannot replace an explicit psychological theory (Kandel, 1999).

### A Partial Mechanistic Explanation—Brain Circuits of Working Memory

One example of a successful, heuristically useful model that can “explain” a mental function by neuronal mechanisms is the model of the working memory and its impairment in schizophrenia (Kendler and Schaffner, 2001; Lewis et al., 2012). Working memory is understood as the transient holding of information ready for further processing like comparison or prediction of upcoming stimuli. Imaging studies in humans as well as electrophysiological experiments in monkeys showed that the temporal structure of neuronal activity of pre-frontal cortical areas correspond to the quality of working memory function. A further analysis of the local brain circuitry by special EEG recordings showed that low gamma oscillations are correlated with impaired working memory (Uhlhaas, 2015). Histological studies indicated that structural deficiencies of inhibitory GABA neurons underlie this dysfunction (Lewis et al., 2012). Finally, genetic studies showed specific genes possibly playing a causal role in the dysfunctional formation of these neurones (Lewis et al., 2012).

This model is a convincing multi-level bottom-up explanation that allows us to relate a clinical syndrome to neurobiology. Heuristically useful computational simulation models could be constructed whereby effects of different neurotransmitters can be explored (Wang, 2010).

## Philosophy

### Summary

A big problem of modern neuroscience is its lack of *reflection*, i.e., the inability to critically ask itself (Bennett and Hacker, 2003). Therefore, the methodology of integrative human neuroscience has to include a discipline specialized on reflection, i.e., philosophy. This would permit neuroscientists to consider often neglected implicit assumptions on whose basis they work.

Particularly, philosophical anthropology supports our view that methodological dualism is inevitable in neuroscience and does not imply any ontological dualism (e.g., between nature and culture; or between brain and body periphery). Phenomenology stresses that the brain cannot be regarded as a machine that “produces” mind, but as a vital component in a highly complex transaction between brain, body, physical and cultural world. Philosophy of science shows that young sciences have yet to learn how to avoid overgeneralizations, absolutistic claims and hyper-constructivist messages (e.g., “there is no world outside the brain”) that dangerously approach solipsism.

### Epistemology and Philosophy of Science

One central question is how neuroscience can obtain knowledge about brain-mind relations. Regarding this, the history of physics might reveal analogies with, but also differences from, the recent history of neuroscience. We know from the history of physics that the ideas are rapidly generalized at the beginning of a new era of research. These generalizations vanish, however, when the euphoria of novelty passes. The more details we learn, the more difficult it will be to tell simple stories with universal claims.

Another lesson from history and philosophy of physics is the necessity of an elaborated theoretical neurobiology that goes beyond pure computation (Edelman and Tononi, 2001; van Hemmen, 2014; Hobson and Friston, 2014). Neither neurobiology nor philosophy of neuroscience is so far developed.

Also, philosophy of physics has learnt to avoid both the Scylla of absolute truth claims and the Charybdis of radical instrumentalism. It rejects the constructivist view that science is but a construction of useful models having nothing to do with “reality”. Rather, theoretical progress is regarded as approximation to the real state of affairs in the world (e.g., Lakatos, 1976; Psillos, 2000). On the other hand, contemporary basic sciences clearly distinguish between truth and explanatory power. Even completely wrong models (e.g., geocentric astronomy) may have a lot of explanatory power within narrow limits (e.g., in map building). A good example in the neuroscience is the well-accepted conception of an EEG signal as created exclusively by gradual post-synaptic potentials of neurons, as if axonal spike potentials do not exist. Moreover, we were able to explain a very broad range of ERP effects on the basis of a very simplified view that only radial cortical dipoles do matter whereas tangential dipoles do not (Kotchoubey, 2006). Unfortunately, views on neuroscience sometimes wildly oscillate between the polar positions directly contradicting each other. On the one hand, there are metaphysical claims that the whole world is merely a construction of the brain (e.g., Revonsuo, 1995a,b; Lehar, 2003). Suddenly, one radical exception is made from this radical anti-realism; namely, the findings of neuroscience are regarded as “objective facts” that are not subjected to any interpretation.

Particularly strong realistic bias exists in discussing specific experimental findings. Thus a significant BOLD response in, e.g., the right temporal lobe during a particular mental task is interpreted as the localization of the corresponding mental function in the right temporal lobe, while in fact, the finding indicates at the very best that the right temporal lobe might be more important for performing this mental task (in the particular conditions) than other brain regions.

A very difficult question is that of the appropriate complexity of theoretical approximations and models of brain activity. While such theories typically simplify the real state of affairs (see EEG and ERP examples above), there is a limit of simplification below which a theoretical model cannot work. It can be particularly questioned whether purely linear models can suffice for adequate description of brain circuits characterized by re-entrance and self-sustainment.

There is another aspect of modeling also related to the issue of models’ veridicality. Neuroscientists not only use models but also typically regard the brain itself as a modeling device, which is then interpreted in a strong anti-realist sense: the brain is said *not* to reflect reality, but to model it (e.g., Metzinger, 2003). This is a profound misunderstanding of modeling process. Modeling is not opposed to veridical representation; rather, models represent real phenomena in a simplified manner, stressing some of their features and ignoring other (“less important”) features (Bailer-Jones, 2009). Each model is a model of something, and thus if we say that the brain builds a model of the self we admit that the self really exists.

### Neurophilosophy and The Conceptual Loop

As we have said above in discussing neuropsychology, the quality of experiments in BACN critically depends on the quality of psychological concepts that undergo neurophysiological analysis. Many basic psychological concepts root in folk psychology or in the philosophical tradition. Generally, the possibilities of neuroscientific experimentation are limited by the conceptual framework provided “top-down”, from philosophy through psychology. For example, many neuroscientists try to find neurobiological foundations of consciousness, but nobody aims at neurobiology of the soul. This is not because some experiment in neuroscience has found out that there is no soul, but rather, because philosophy, cognitive psychology and the present-day common sense assume that consciousness is a more useful concept than soul.

However, this is not the whole truth. The fact that concepts strongly determine the course of experimentation, does not mean that the experimenters are enclosed in the prison of given concepts. Thus psychological experiments to such phenomena as attention and memory have changed the content of these (primarily folk) concepts. Likewise, psychological studies to philosophical concepts such as intentionality are able to modify these concepts (e.g., Iijima and Ota, 2014). The same is true for the experiments in neuroscience. A careful examination of brain activity related to self-linked notions and to conscious processes can even result in a revision of some philosophical models of self and consciousness (Northoff, 2013). The conceptual relationships between philosophy, psychology and neuroscience are not a one-way road, therefore, but rather a closed loop. On the one hand, philosophical concepts define the primary frame, in which empirical (psychological and neurobiological) studies are designed; on the other hand, the results of these studies may change the meaning of the concepts.

### Phenomenology

Many mental concepts were coined in the context of phenomenological philosophy (e.g., consciousness or the self), even though the later psychology used these notions differently from their phenomenological basis. Still a philosophical reference is useful in clinical psychiatry (Jaspers) as many psychiatric disorders involve a disturbance of the basic experience of the person as being situated in the world.

A mind without brain is nothing; but the brain is not everything for the mind. The phenomenological approach emphasizes the *embodied, embedded, enacted and extended* nature of all human experience. “Embodied” means that the brain does not generate consciousness as being in a NaCl solution but only in the interaction with bodily periphery (including the peripheral nervous system, but also inner organs, skin, and the motor apparatus). This brain-body system is further “embedded” into the environment. “Enacted” means that neither the brain nor mind are pure information processing machines but work in a continuous interaction with the environment, in which the organism plays the active role pursuing its goals notwithstanding environmental disturbances. This, in turn, implies that mental processes cannot be conceived separately from bodily processes (“extendedness”). After the pioneer publication of Clark (1997), which explicitly referred to the phenomenological tradition, the idea of embodiment became one of the leading concepts of modern cognitive psychology. Importantly, embodiment does not mean simply the fact that the brain is “inserted” into a body and “constrained” by bodily factors, but, rather, that the interaction between the brain and the body is the essential constituting condition sine qua non mental activity (Tschacher and Bergomi, 2011).

Whereas phenomenology has traditional relations with psychopathology (Jaspers, 1913/1997), links with experimental psychology remain weak. The anti-atomistic stance and the emphasis on the embodied/embedded nature of mind raise strong associations with ecological psychology (e.g., Kadar and Effken, 1994). Both ecological approach (Gibson, 1979) and phenomenology of perception (Merleau-Ponty, 1945/1965) have influenced contemporary theories of perception and action (e.g., Clark, 1997; Noe, 2005).

### Philosophical Anthropology

Human beings are conditioned by both nature and culture. For centuries, philosophers regarded man—the only creature able to reason and language—as the highest being. The present interest in the animal heritage of man, making biology a leading discipline, is justified. This interest in the facts of natural science, however, does not imply that everything in humans can be explained by natural science. The facts of culturally determined neuroplasticity demonstrate that humans remain cultural beings even as biological objects (i.e., in their anatomy and physiology). This is true that humans should be studied by natural sciences. But this is not the same as to say that humans can only be conceived of in terms of natural sciences. Rather, a double perspective should be defended that goes beyond the opposition between a fundamental anti-naturalism, on the one hand, and a reductive naturalism, on the other hand.

Biological (including neurobiological) approach do not contradict humanity. Biology and culture are not additive parts that result in a human being as their sum, but, rather, two overlapping perspectives. Human culture also has its biological basis. Naturalism does not question the universality of human rights, but even the opposite is true: understanding of natural human conditions may help to experience empathy with the fragility and finiteness of human existence. Just to remember that a brief carelessness during car driving, or a small fishbone in the throat, may end a human life. Neither reason nor culture as such can fundamentally change this natural endangerment of mankind: as a “being to death” (Heidegger, 1927/2010), the human being cannot completely rearrange its biological constitution.

This fact indicates the problem of a reductive program that aims to translate ordinary expressions of human language about our mental events and emotions in expressions of neurosciences. If facts and findings of neurosciences rightly question some old-fashioned forms of rationality, this does not mean that we should stop saying things like “I love you”. Anyway, it is a cultural tradition which allows speaking about the results of natural science. It is not science itself which speaks but we as cultural beings do it. The legitimate comparison between neurophysiological processes and such phenomena as consciousness and emotions does not mean that consciousness and emotions can be conceived of, or translated, in the terms of brain responses.

The attempts to naturalize anthropology are very old. The real impact of new findings of modern neuroscience should, therefore, be kept apart from the ancient metaphysical claims of materialism and determinism. These claims are mostly of philosophical, not scientific, character and possess astonishing similarity in the 18th, 19th and 21st centuries. We have, therefore, to distinguish between new scientific concepts and a new brain ideology. When results of a particular experiment are used to justify a radical change in cultural practice, this is not only an undue overgeneralization but also a crude category error: even a correct statement about what is cannot tell us about what ought to be. The neurosciences would be on a better way if their concepts are kept within the reflected limits of knowledge. It is a task of cultural interpretation to clarify what follows from the results of neuroscience and what does not. It is a contradiction to say that neuroscience is the best approach to our mental life and, at the same time that it is not about our mental life in the terms we normally use.

Science, and particularly natural science, views the world in a methodological way. This is not only correct but even constitutes the essence of scientific enterprise. However, this also yields a permanent discrepancy between daily and scientific approaches to the mind. As a person I am interested in all the details of my own love to a particular person, but not very much in the general functional mechanisms of love in humans. It is not clear* a priori* whether science is suitable to answer questions of our practical relevance.

Modest *scientific realism* (Psillos, 2000), which we mentioned in Section “Epistemology and Philosophy of Science” and which maintains that scientific theories can approach reality, should not be confused with *scientism* claiming that science is the only (or the main) way to know reality. In its neurobiological version, scientism asserts that neuroscience is in a privileged position to tell us what the human mind really is as compared with psychology, philosophy, art, not to speak of religion and the common sense. However, while scientific theories help us to understand reality, this does not mean that these theories *are* reality. Science and technology are ways of viewing the world among others, and the world is already “erschlossen” (which means that every understanding begins with a pre-conception; Heidegger, 1927/2010). Reality itself is silent; we are the ones who talk about it.

### Ethics: The Power of Interpretation and the Neglect of Subjectivity

Several ingenuous publications have been devoted to numerous ethical problems of neuroscience (e.g., Levy, 2007; Illes and Sahakian, 2011; Chatterjee and Farah, 2013). In the present text, we only briefly indicate those of the problems that have immediate methodological implications. Neurosciences do not merely present pure facts and theories of the brain but have consequences for many practical attitudes, e.g., medical treatment. Scientists often follow the program of searching for facts and avoiding errors, fiction and ideology. There are reasons to do so. Nevertheless, there are no “bare facts” without interpretation. Open to interpretation in science are e.g., the way you use your instruments, the way you think your methods lead to the truth, the way you connect your findings with other findings, the way you put your results into a (more or less practical) context. In sum, every talk of facts has a history of constitution which is relevant for the truth of your scientific work. The power of interpretation leads to an indirect justification of pluralism in science.

For example, scientism in the practical medicine can lead to physicians becoming more and more specialized scholars, who often cannot talk to their patients as persons. But the philosophical definition of the human nature has immediate practical consequences. A physician who regards his patient, like some neuroscientists suggest to do, as a complex neural network with skin and bones around it, treats this patient differently from that physician who regards the same patient as a person (e.g., Stier et al., 2014).

Like every mental phenomenon, psychiatric (i.e., pathological) phenomena can have more or less specific brain mechanisms. Therefore, it is not incorrect to regard psychiatric problems as brain dysfunctions. This does not imply, however, that this neuroscientific approach is* a priori* better than other approaches, e.g., based on daily experience or on experimental psychology. Therefore, we can use chemical medicaments to cure mental disorders, but we cannot know without an empirical demonstration that such medicaments are better than, e.g., personal or psychological treatments.

## Systems Science and Theoretical Perspectives in Neuroscience

### Summary

Systemic approach can integrate theories of different levels in so far they tackle common problems such as non-linearity, self-organization or complexity that can emerge at any (biochemical, neurophysiological, psychological, social) level. Mathematical models are useful tools to represent highly complex processes. However, several factors considerably reduced the integrating power of this approach in the past and still limit its application in present.

One of them is precocious mathematization. Mathematical formulations and computations do not suffice to “understand” neural phenomena. For neuropsychological modeling more conceptual work has to be done. Another negative factor is theoretical overgeneralization. The ability to think in general terms is a big advantage of systems sciences. This advantage can help to overcome the limitations of narrow disciplines. On the other hand, the advantage easily flips over into a disadvantage when the terms and theories are *too* general. Finally, the problem of the optimal complexity of systemic models still waits for solution.

### Characteristic Features of System Science

Interdisciplinary integration entails a meta-scientific perspective. Concepts of System Science, such as balance, stability, feedback loop, self-organization, etc., which are neither material nor mental, nor social, are needed for rendering neuroscientific descriptions complete. They can help to overcome particularities of disciplinary views.

The general features of sciences as listed above are also valid for Systems Science. The *objects* of Systems Science are systems regardless of their physical realization (including symbolic systems). Examples of typical *concepts* have been given above. Regarding *methods*, systemic models are often presented in mathematical or graphical language. Both classical differential equations and graph theoretical formulations are employed (e.g., Kitano, 2002), as well as computer-based simulated models. The models are developed in close interaction with empirical observations. Finally, *theories* used in Systems Science (e.g., catastrophe theory, chaos theory, complexity theory) provide a formal approach to understand the behavior of systems on a “supradisciplinary” epistemic level. However, in many cases computer simulations do not result in an elaborated *explanatory* theory but only in *exploratory* models, whose epistemic status remains the matter of debate.

### Historical Background

Traditionally, classical *biocybernetics* was used in a higher-level functional analysis of the brain in order to describe and analyze global and local control loops. This approach on the early stage of theoretical brain research was mainly focused on filter theory, theory of regulation, flow-equilibrium and information theory (Wiener, 1948; Ashby, 1956; von Bertalanffy, 1968; Arbib, 2002). Later on, the concepts of artificial intelligence, especially the concepts of artificial neural networks, were developed to model information processing in the brain (Arbib, 2002). Artificial neural network models were a breakthrough in theory building and modeling. They were applied to the issues of pattern recognition and specific hardware realizations of this process.

Most theoretical concepts in neuroscience are closely related to different theoretical paradigms that popped up since World War II. Thus catastrophe theory helped to understand jumps in behavior trajectories of living systems, and chaos theory formalized irregular complex behavior patterns. In consequence, theoretical neuroscience and computational neuroscience are fields that are characterized by a high degree of mathematical descriptions of brain processes (Dayan and Abbott, 2001). The physical model of Hodgkin-Huxley, presented in a form of an elegant mathematical equation that describes, explains and predicts membrane potential of a neuron by the action of ion channels, is possibly the best known theoretical model in neuroscience (Hodgkin and Huxley, 1952). Additionally, the new wave of concepts of self-organization was started (e.g., Haken, 2002; Liljenström, 2010). However, mathematical models should be based on an appropriate conceptual apparatus (van Hemmen, 2014). The credit of Isaac Newton was not only that he found the nice formula *F = ma*, but that he introduced the fundamental notions of force, mass and acceleration. Without such fundamental notions, mathematic models in theoretical neuroscience can, in the worst case, become just symbolic games.

The early models were criticized for the limited correspondence to the real structure of the brain. To respond to this critique, Computational Neuroscience tried to avoid mathematical modeling that had no empirical correspondence (Dayan and Williams, 2006). Therefore, this field aims to be based on experimental data and uses mathematical tools in order to describe electrophysiological and biochemical phenomena such as neural signaling (Dayan and Abbott, 2001).

Other authors (e.g., Markram, 2006) claim that “data-driven theories” should be constructed, meaning that any computational model that allows calculation of neuronal phenomena is appropriate. However, the complexity problem of data sets diverts the attention from conceptual issues to formal techniques that intend to identify latent structures in data sets such as machine-based algorithms.

### The Need for Systemic Conceptual Frameworks

Above we mentioned some examples of mechanistic explanation in neuroscience. However, the power of such explanations drops with the number of feedback loops within a system. Sarter et al. (1996) showed on the example of a heating device that introducing even a single and simplest feedback loop dramatically complicates a mechanistic explanation, because the relationship between the activity of the heater and the room temperature becomes non-linear. The brain, with its millions of re-entrant (Edelman, 1989) connections is a non-linear device par excellence. How mechanistic explanations can work in such an ocean of non-linearity remains unclear.

Furthermore, our positive experience with mechanistic explanations is based on their ability to disclose part/whole relations. Such explanations demonstrate how macro-phenomena (e.g., field potentials or BOLD responses) can be reduced to, and thus explained by, microscopic electric (local PSPs) and biochemical processes. However, there is no reason to regard the brain/mind relation as a part/whole relation. The brain is not a part of the mind. The relation between the firing of single fibers and the scalp-recorded EEG response to a nociceptive stimulus is a typical part/whole relation, but it is clearly different from the relation between the firing of C-fibers and the subjective feeling of pain.

It should be stressed that the above factors (e.g., non-linearity) still put serious limits on mechanistic explanations, even in the case when such explanations remain purely correlational and do not imply any causality (Craver and Tabery, 2015). Even if non-linear complexity does not completely exclude mechanistic explanations, it complicates them anyway. The “atomistic approach” is nothing but the reduction of the behavior and functions of a whole to the properties and causal activities of its parts, or causal components, and hence it does not differ from mechanistic explanation. Therefore, the atomistic approach in neuroscience, which limits its scope of attention to the internal events (the state and the dynamics of physiological/molecular components of a complex system), should be, if not replaced, at least complemented and counteracted by those views that strongly emphasize embodiment, system-environment interactions and closed-loop perception-action coupling (i.e., ecological, phenomenological and kinesiological approaches), armed with the appropriate theoretical arsenal including chaos theory and catastrophe theory. From our viewpoint, systems methodology is the only one that possesses the appropriate theoretical and conceptual apparat for integration of these views, still regarded as separated from each other. Also the philosophical problem of the part-whole relation is a basic issue of systems science, as it was already discussed by its founders (e.g., von Bertalanffy, 1968).

Another set of methodological problems are related to *partial models* of neural or molecular brain circuits, which are increasingly used to explain clinical symptoms. The models use *wiring diagrams* representing the mode of action of the respective elements such as excitation or inhibition. Such diagrams can be classified as “qualitative models”, on whose basis exploratory mathematical models can be constructed. A model will, then, be used to deduce an experimental design, and this experiment will provide data to be integrated in the model that can be tested again and modified according to the data. This procedure, already developed in the molecular systems biology, can be transferred into neuroscience.

Therefore, a field of theoretical neuroscience appear necessary that works with central systemic concepts such as those listed above. However, at least two important questions remain open. One of them is that of the optimal complexity of appropriate models. For instance, the heuristic value of complex models containing hundreds or thousands of excitatory and inhibitory units seems questionable. What are comparative advantages of simple vs. complex models (Herz et al., 2006)? What should be a criterion of the optimal complexity? What kind of heuristic function the mechanistic models should have (Bechtel and Abrahamsen, 2005)? What kind of heuristic value do graphical models have (Abrahamsen and Bechtel, 2015)?

Another, structurally similar question is that of optimal level of generality of systemic concepts. Above we sufficiently praised the ability of systems theory to formulate concepts overcoming the narrow limits of disciplines and particular approaches. However, the other side of this generality is the use of concepts that are too broad to explain brain and mind as they describe any kind of system. Thus such concepts as self-regulation, self-organization, feedback, bifurcation point, etc. are applicable to objects that have neither nervous system nor mental processes. The need for systemic concepts of the exactly appropriate (not too narrow, not too broad) level of generalization has only recently been realized, and some progress on this way can already be observed (e.g., Jordan, 2003; Bruineberg and Rietveld, 2014; Northoff, 2014a; Beran, 2015).

## Conclusions and Perspectives

The simple hope that more data generated by better techniques would necessarily result in a better understanding of brain and mind underestimates the actual methodological problems. This underestimation has serious negative consequences for both basic and clinical neuroscience. What brain research primarily suffers from is not the insufficient technology but the lack of concepts bridging the explanatory gaps between different levels of brain activity, and between brain events and mental events. It becomes increasingly accepted that a simple localization strategy should be supplemented by basic network conceptions of brain functions. This network turn should be accompanied by similar network conceptions in psychology and psychopathology. For this reason a conceptual framework of psychology should be revisited and a systemic perspective should be developed. The concepts of general systems theory may provide a fruitful basis for further whole-system research. This does not implicate a simple return to the approaches of artificial intelligence or of neuro-informatics. Neither of the systemic approach as proposed here should be conceived of as dominated by mathematical modeling. Rather, exploratory computer-based modeling in service of conceptual models can be seen as an important way to enhance our understanding of the interplay of emotions, cognition and behavior.

If a systemic psychology is developed, a co-evolution of theoretical models in neurobiology and mind sciences may become possible. However, this development requires philosophical reflection, particularly concerning the brain-mind relations. A *methodological parallelism* appears to be a pragmatically appropriate position that does not prefer any ontological position. For this new project of integrating neuroscience, we assume that a homomorphism of methods, concepts, models and theories is necessary. In this development, philosophy and systems science should play an important part for integrating the various methodological approaches in neuroscience.

## Author Contributions

BK: final version of the MS, concepts to the Sections “Psychology”, “Brain-Mind Studies” and “Systems Sciences”. FT: the main idea of the MS, particularly concepts to the Introduction and General Conclusion, as well as the Sections “Psychology”, “The Structure of the Field of Neurosciences, and Systems Sciences”. HAB: contribution to electrophysiological and molecular-physiological aspects of the MS. TB: contribution to the Section “Brain-Mind Studies”. AD and TH: contribution to the Section “Neurobiology”. TF: subsection Phenomenology. FH: contribution to biochemical and genetic aspects of the MS. HH: main concepts of the Section “Philosophy”. GN: contribution to the Sections “The Structure of the Field of Neurosciences” and “Psychology”. IR: contribution to ethical aspects of the MS. SSchleim: contribution to social aspects of the MS. SSellmaier: contribution to the sections “Philosophy” and “Brain-Mind Studies”. LTVE: contribution to clinical aspects of the MS. WT: contribution to the Section “Systems Science”.

## Conflict of Interest Statement

The authors declare that the research was conducted in the absence of any commercial or financial relationships that could be construed as a potential conflict of interest.

## Footnotes

^1^http://www.jneurosci.org/site/misc/about.xhtml

^2^An example from Kotchoubey (2007) shows how difficult this venture is: if, e.g., we say “my prefrontal networks process complex information” instead of, I think, this would not result in any conceptual reduction, because the term “my” in the latter expressions still remains a mental concept indicating the existence of some “me”.

^3^“Distribution requirements are derived from the mathematics, chemistry and physics departments; and electives can come from departments such as anthropology, philosophy and history” (http://www2.binghamton.edu/integrative-neuroscience/).

^4^“A unique aspect of the integrative neuroscience major is the presence of three tracks (cell and molecular, cognitive and systems and computational), each focusing on a specific aspect of neuroscience” (http://www.fordham.edu/academics/programs_at_fordham_/integrative_neurosci/).
